# Simple *Trans*-Platinum Complex Bearing 3-Aminoflavone Ligand Could Be a Useful Drug: Structure-Activity Relationship of Platinum Complex in Comparison with Cisplatin

**DOI:** 10.3390/ijms21062116

**Published:** 2020-03-19

**Authors:** Małgorzata Fabijańska, Magdalena Orzechowska, Agnieszka J. Rybarczyk-Pirek, Justyna Dominikowska, Alicja Bieńkowska, Maciej Małecki, Justyn Ochocki

**Affiliations:** 1 Department of Bioinorganic Chemistry, Medical University of Lodz, 1 Muszynskiego St., 90-151 Łódź, Poland; 2 Department of Applied Pharmacy, Medical University of Warsaw, 1 Banacha St., 02–097 Warsaw, Poland; magdalena.orzechowska@wum.edu.pl (M.O.); abienkowska@wum.edu.pl (A.B.); mmalecki@wum.edu.pl (M.M.); 3 Theoretical and Structural Chemistry Group, Department of Physical Chemistry, Faculty of Chemistry, University of Łódź, Pomorska 163/165, 90-236 Łódź, Poland; agnieszka.rybarczyk@chemia.uni.lodz.pl (A.J.R.-P.); justyna.dominikowska@chemia.uni.lodz.pl (J.D.)

**Keywords:** anticancer drugs, coordination compounds, gene expression, platinum, structure–activity relationships

## Abstract

Following previous studies devoted to *trans*–Pt(3-af)_2_Cl_2_, in this paper, the molecular structure and intermolecular interactions of the title complex are compared with other cisplatin analogues of which the crystal structures are presented in the Cambridge Structural Database (CSD). Molecular Hirshfeld surface analysis and computational methods were used to examine a possible relationship between the structure and anticancer activity of *trans*–Pt(3-af)_2_Cl_2_. The purpose of the article was also to investigate the effect of hyperthermia on the anticancer activity of cisplatin, cytostatics used in the treatment of patients with ovarian cancer and a new analogue of cisplatin-*trans*–Pt(3-af)_2_Cl_2_. The study was conducted on two cell lines of ovarian cancer sensitive to Caov-3 cytostatics and the OVCAR-3 resistant cisplatin line. The study used the MTT (3-(4,5-dimethylthiazol-2,5-diphenyltetrazolium bromide) cell viability assay, LDH (lactate dehydrogenase), and the quantitative evaluation method for measuring gene expression, i.e., qPCR with TagMan probes. Reduced survivability of OVCAR-3 and Caov-3 cells exposed to cytostatics at elevated temperatures (37 °C, 40 °C, 43 °C) was observed. Hyperthermia may increase the sensitivity of cells to platinum-based antineoplastic drugs and paclitaxel, which may be associated with the reduction of gene expression related to apoptotic processes.

## 1. Introduction

Ovarian cancer remains the most serious cause of death among gynecologic cancers. Cancer is a serious medical and social problem in Poland and in the world, despite progress in diagnosis and chemotherapy. In Poland, ovarian cancer occupies the fifth position among malignant tumors in women after breast, lung, stem of the uterus, and colon cancers. Ovarian cancer in the death structure is in fourth position after lung, breast, and colon cancer. Every year, more than 3600 women are diagnosed with a malignant ovarian cancer, and it is also the cause of deaths of 2600 women [1]. In 2018, about 22,280 women were diagnosed with ovarian cancer in the United States, and about 15,500 died because of this disease, which is the most common cause of fatal gynecologic tumours in developed countries [2].According to data, in 2017 there were as many as 3775 new cases of ovarian cancer diagnosed in Poland [3]. According to the American Cancer Society data from 2020, estimates of ovarian cancer in the United States are about 21,750 women will receive a new diagnosis of ovarian cancer, about 13,940 women will die of ovarian cancer [4]. The treatment of patients with ovarian cancer, regardless of the stage of clinical advancement, involves surgical resection of neoplastic lesions and the implementation of adjuvant chemotherapy based on platinum derivatives and taxoids. This treatment allows for complete responses in approximately 75% of patients. However, three quarters of them relapse, and five-year long-term survival is only seen in 25% of patients [5,6,7]. There are many chemotherapeutic agents used to treat ovarian cancer, depending on the type, grade, stage of the disease, and the health of the patient. Meta analyses of randomized clinical trials have shown that platinum compounds (cisplatin/carboplatin), are the most active agents in ovarian cancer [8]. The inclusion of cisplatin in the chemotherapeutic regimen for advanced ovarian cancer proved to be a major landmark. Cisplatin binds to nuclear DNA leading to interference with transcription and/or DNA replication and eventually cell death induced by cell repair machinery. A Cochrane review and meta-analysis confirmed a two- and five-year survival advantage in women with advanced stage epithelial ovarian cancer who were given platinum-based combination chemotherapy compared with those given combination therapy lacking platinum.

Cisplatin is commonly used to treat solid tumours, e.g., ovarian and breast cancer. It is one of frequently used medications in anticancer therapy. Unfortunately, like any cancer medication it causes a number of side effects, e.g. nephrotoxicity. Combinations using platinum(II) compound with documented anticancer properties is a new concept in the search for new ways to treat ovarian cancer. Chemoprevention with the use of natural or synthetic compounds, such as flavonoids, may inhibit or prevent cancer progression, which is why it has become an attractive strategy to fight cancer [9].

Over the past 20 years, there were reports showing that replacing the amino group with the appropriate ligand in the transplatin molecule increases the antitumour activity of the compound [10,11]. *Trans*-Pt(3-af)_2_Cl_2_, the coordination complex of platinum(II) with 3-aminoflavone, is a combination of platinum(II) compound with flavonoid ligands. The compound showed cytotoxicity for many tested cancer cell lines. Moreover, it proved to be much less toxic for normal lymphocytes in comparison to cisplatin, which decreases the number of potential adverse effects [12].

Hyperthermia can present a hope for patients suffering from ovarian cancer. In oncological therapy, hyperthermia is defined as a controlled technique for raising temperature in the area of neoplastic lesions to destroy their cells or to inhibit their growth. The cytotoxic effect is revealed in the temperature range of 40–43 °C [13]. Hyperthermia is often combined with radiotherapy and intravenous and intraperitoneal chemotherapy [14,15,16,17,18,19]. The combination of elevated temperature and radiotherapy results in, for example, inhibiting the repair of sublethal DNA defects that are created after irradiation, destroying cells in the radioresistant phases of the G1 and S cell cycle and increasing tumour cell oxygenation.

Intraperitoneal perfusion chemotherapy under hyperthermia (HIPEC) is a procedure that consists of cytoreductive surgery (weight reduction surgery cancer) and peritoneal chemotherapy administration in hyperthermia. The HIPEC procedure is used in selected cases of patients with recurrent ovarian cancer with intraperitoneal dissemination and in other cancers accompanied by dissemination [20,21]. A very important condition of the HIPEC procedure is the removal of macroscopic focia tumor larger than 5mm in diameter, and that relapse occurs at least after one year from primary treatment due to platinum sensitivity which is associated with better response to the HIPEC procedure. Based on the initial experience of the team at Śpiewankiewicz, management can say that the HIPEC procedure is valuable in supplementing the recognized methods of oncology, the condition is appropriate, multidisciplinary qualification for the procedure [22]. 

The HIPEC procedure involves administration infusion fluids into the peritoneal cavity and raising the temperature of organs within it 41–42 °C. The advantages of the HIPEC method are as follows: damaging effect on cells cancer, increasing the penetration of cytotoxic drugs, potentiation of action anticancer cytostatic drugs [23]. 

The HIPEC procedure is used several anticancer drugs: doxorubicin, cisplatin, carboplatin, mitomycin C [24]. For ovarian cancer, the HIPEC procedure is experimental because it doesn’t randomized trials were conducted. In ovarian cancer, the procedure is used cisplatin, doxorubicin and taxanes.

Following our previous studies on *trans*–Pt(3-af)_2_Cl_2_, in this paper, the molecular structure and intermolecular interactions of the title complex are compared with other cisplatin analogues of which the crystal structures are presented in the CSD. Possible relationship between the structure and anticancer activity of *trans*–Pt(3-af)_2_Cl_2_ (in comparison with cisplatin and other platinum compounds) was examined. The article shall examine the effect of hyperthermia on the anticancer activity of cisplatin and cytostatics used in the treatment of patients with ovarian cancer and a new analogue of transplatin with 3-aminoflavone *trans*–Pt(3-af)_2_Cl_2_. The study was conducted on two cell lines of ovarian cancer: sensitive to Caov-3 cytostatics line and the OVCAR-3 resistant cisplatin line.

Cisplatin is a medicine that is often used in anticancer therapy, however its side effects are serious, which is why new compounds, therapies that would be not so heavily affected by adverse reactions, are still being sought. Recently, a lot of attention is focused on searching for new analogues of already existing drugs or replacing substituents with other ligands. The example of *trans*–Pt(3-af)_2_Cl_2_ proves that even an inactive anti-neoplastically transplatin compound with another ligand may have an anti-neoplastic potential. New compounds, but also new therapies, constitute an attractive goal for research. In this article, the influence of oncological hyperthermia on the tested compounds was investigated. 

## 2. Results

### 2.1. Structural Analysis

The *trans*-platinum(II) complex of 3-aminoflavone (*trans*–Pt(3-af)_2_Cl_2_), similarly to cisplatin, displays significant cytotoxic activity. It belongs to a relatively small group of *trans* platinum complexes displaying cytotoxic properties. The crystal structure of the title compound was determined by X-ray methods [12].The molecular structure of the title complex consists of the platinum cation surrounded by two 3-aminoflavone ligands in their neutral form and the two chloride anions. Coordinating nitrogen and chlorine atoms are arranged in a slightly distorted square with Cl-Pt-N angles values equal to 87.3(2) and 92.7(2) Å. It is worth noting that *trans*-Pt(3-af )_2_Cl_2_ complex compound crystallises in the triclinic *P*$\bar{1}$ space group with platinum atom located in special position in the crystal unit cell, in the inversion point. As a result, its molecular structure shows local *C_i_* symmetry. The benzopyrane fragment of the 3-aminoflavone ligand is essentially planar and forms the dihedral angle of 78.1(2)° to basic square plane.

Following the previous studies devoted to *trans*–Pt(3-af)_2_Cl_2_, in this paper the molecular structure and intermolecular interactions of the title complex are compared with other cisplatin analogues of which the crystal structures are presented in the CSD (Cambridge Structural Database).

**Figure 1:**
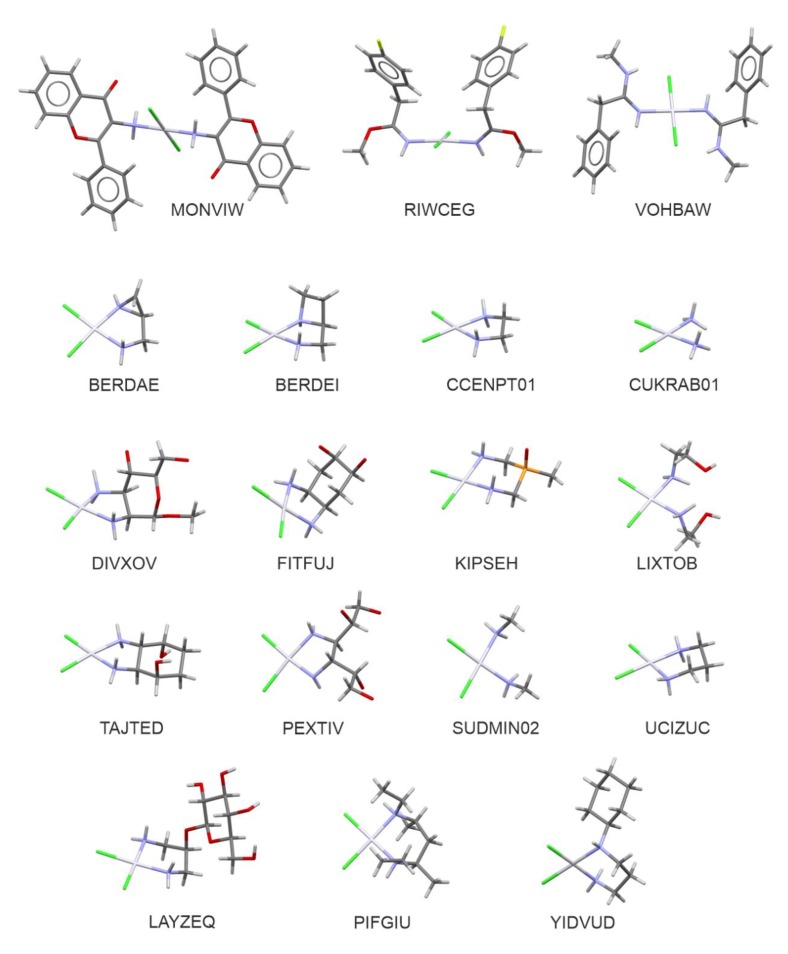
Molecular structures of biologically active analogues of cisplatin and transplatin (with the CSD refcodes).

The search of the CSD revealed 219 crystal structures of four-coordinated platinum(II) complexes with two ammine-resembling and two chlorido ligands. All of these complexes displayed planar square coordination around the central Pt atom. With some more limiting criteria (see the methodology section for details) this number was further limited to 131, including 83 examples of crystals with Pt(NH=R)_2_Cl_2_ moiety, 47 with Pt(NH_2_R)_2_Cl_2_ one, and the *cis*-diamminedichloroplatinum(II) dimethylformamide solvate [25] (reference code: CUKRAB01). In this group, there are 18 crystal structures recorded in the CSD as compounds with the confirmed biological activity of cytotoxic, antitumor, or anticancer properties. It is important to stress that the majority of the compounds displaying such biological activity represent a class of chelatecomplexes of *cis*-configuration: BERDAE, BERDEI [26], CCENPT01 [27], DIVXOV [28], FITFUJ [29], LAYZEQ [30], LEFFAD [31], LIXTOB [32], PEXTIV [33], PIFGIU [34], SUDMIN02 [35], TAJTED [36], TUPQIE [37], UCIZUC [38], and YIDVUD [39]. In contrast, the title *trans*–Pt(3-af)_2_Cl_2_, recorded with MONVIW reference code, together with RIWCEG [40] and VOHBAW [41] structures, belong to a group of only a few *trans* platinum complexes showing cytotoxic activity. Figure 1 presents molecular structures of the compounds listed above.

A comparison of geometric parameters characterizing structural arrangement around the platinum center in the investigated group of complexes is presented in Table S1. The *trans* complexes can be easily recognized by values of Cl-Pt-Cl and N-Pt-N angles which are close (RIWCEG) or equal to 180° (the latter situation resulting from the symmetry of complexes with Pt atom in the inversion center in MONVIW and VOHBAW crystal structures). In the *cis* complexes the values of the corresponding angles are, as expected, very close to 90°, but Cl-Pt-Cl angles display rather larger mean values (from 90.8° to 94.8°) than the values of N-Pt-N angles (from 82.7° to 93.0°). Interestingly, in the case of the *trans* complexes the values of Cl-Pt-N angles are somewhat smaller (with the mean value of 86.7°) than the values observed for the *cis* complexes (90.1°). These differences may result from differences in platinum bond lengths (from 2.298 Å to 2.325 Å for Pt-Cl and from 2.005 Å to 2.085 Å for Pt-N), as well as from the chelate nature and steric hindrance of organic ligands. For *trans*–Pt(3-af)_2_Cl_2_ the corresponding valence angles around Pt atom are within the presented ranges, but bond distances evidently vary from the mean values—the Pt-Cl contacts are relatively shorter, whilst the Pt-N bonding distances are relatively longer as shown in Supplementary Table S1.

According to the literature, the first step of cisplatin activation is connected with platinum hydration and the replacement of one of the chloride ions with the water molecule [42]. According to the well-known statement of Bürgi and Dunitz [43], each crystal structure may be treated as a frozen state corresponding to a particular stage of the reaction. From this point of view a detailed analysis of intermolecular interactions around the platinum atom seems to be of great importance. In the present paper the molecular Hirshfeld surfaces were calculated for all the complexes from the analyzed group of crystal structures. Additionally, the values of d_norm_ parameter were calculated and analyzed to indicate the most important intermolecular interactions.

**Figure 2:**
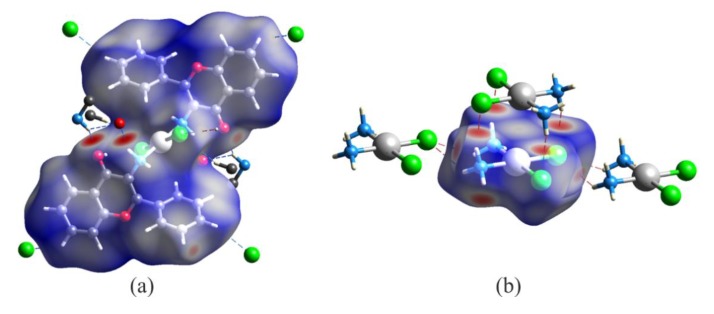
Molecular Hirshfeld surfaces of *trans*-platinum(II) complex of 3-aminoflavone (MONVIW) (**a**) and *cis*-diamminedichloroplatinum(II) complex (CUKRAB01) (**b**) mapped with the d_norm_ parameter. Red areas correspond to very short and important stabilizing intermolecular contacts characterized by negative values of d_norm_.

In the crystal structure of *trans*–Pt(3-af)_2_Cl_2_, the molecules are linked by intermolecular N-H…O hydrogen bonds and organized into chain motifs. The Hirshfeld surface analysis results presented graphically in Figure 2a, indicate that the nitrogen atom from the N-H…O interaction and the chlorine atom from the C-H…Cl- interaction are both the most active sites of the molecule (red areas in Figure 2a). The obtained picture can be compared with the one obtained for the *cis*-diamminedichloroplatinum(II) complex. Interestingly, in this case, apart from N-H…Cl- hydrogen bonds forming chain motifs, the molecules also interact with each other in a plane-to-plane style (Figure 2b). Such a close packing is also stabilized by intermolecular N-H…Cl- hydrogen bonds and results in a situation resembling stacking molecular arrangement. This results with Pt…Pt distances of 3.407(2) Å that are somewhat shorter than the sum of corresponding van der Waals radii (3.44 Å) [44]. In general, the direction perpendicular to the plane of the complex (defined by positions of Pt, N and Cl atoms) seems to be very important from a viewpoint of the previously mentioned mechanism of cisplatin activation. Interestingly, a similar plane-to-plane arrangement of Pt, Cl and N atoms was found for other complexes, namely: CCENPT, FITFUJ, PEXTIV, TAJED, and UCIZUC among the cis isomers and RIWCEG among the trans ones. When comparing Hirshfeld surfaces of all the analyzed complexes (see Table 1) one can notice a relatively small percentage of platinum intermolecular contacts – usually below 5% (apart from a non-substituted cisplatin solvate: CUKRAB01). Interestingly, in this group, the contribution of the Pt…Pt interactions changes from 0 to 60%, but the dominant contribution is observed for Pt…H contacts (from 40 to 100%). It seems that the latter results from various types of D-H…Cl- stabilizing hydrogen bonds (where D – denotes any kind of a hydrogen bond donor) between ligands of neighboring complexes, similarly to the cisplatin CUKRAB01 structure. It is also important to point out here that the total contribution of Cl…H contacts in all the investigated crystal structures is meaningful and changes from 8.5% up to 43.5% (mean value for the *cis* complexes is about 28%).

Summarizing the results of Hirshfeld surface analysis, it may be concluded that the areas above and below the platinum atom (with respect to the plane of the complex) play important role in stabilization molecular packing in the crystal state. Moreover, from the point of view of the cisplatin activation mechanism, the obtained results suggest that platinum atom could be rather easily available for water molecules and the first step of hydration process may be connected with formation of O-H…Cl^−^ hydrogen bonds between the water molecule and the chlorido ligand.

### 2.2. Computational Results

**Figure 3:**
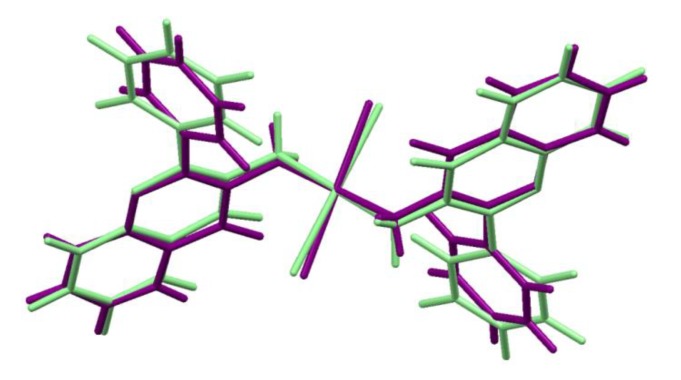
Overlaid geometries of *trans*-Pt(3-af)_2_Cl_2_ molecules: present in the crystal structure (purple) and optimized at the B3LYP/def2-TZVPP level (light green).

The differences between the geometry of *trans*-Pt(3-af)_2_Cl_2_ molecule in the crystal structure and its geometry optimized in vacuo are shown schematically in Figure 3. The numerical data for the chosen geometrical parameters for both geometries are collected in Table 2. If one considers the platinum atom vicinity (the most important from a viewpoint of cytotoxicity), some structural changes are visible.

In the case of the geometry obtained at the B3LYP/def2-TZVPP level of theory, the Pt-N distance (2.100 Å) is elongated with reference to the crystal structure (2.064(5) Å). Such elongation also takes place in the case of the Pt-Cl bonding. The bonding distance is equal to 2.340 Å for the optimized structure and 2.298(1) Å in the crystal structure. Similar elongation of the Pt-N and Pt-Cl distances in the case of optimized structures was previously observed for cisplatin [45] and its analogues [46]. Interestingly, the N-Pt-Cl angle value is greater for the optimized geometry (95.7°) than for the crystal structure (92.7(1)°), indicating that it is rather the crystal packing that makes the Pt atom surrounding closer to the square arrangement. 

**Figure 4:**
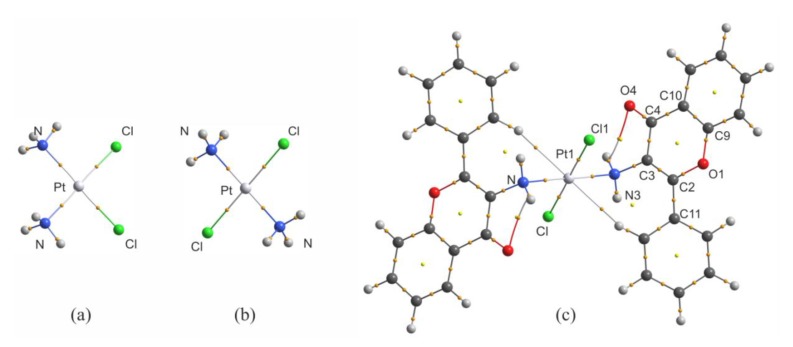
Molecular graphs of cisplatin (**a**), transplatin (**b**) and *trans*-Pt(3-af )_2_Cl_2_ (**c**) calculated at the B3LYP/def2-TZVPP level.

To discuss the results of QTAIM analysis for *trans*-Pt(3-af)_2_Cl_2_, it is worth comparing the QTAIM parameters with the results obtained for cisplatin (of high cytotoxicity) and transplatin (of low cytotoxicity). This is important due to the fact that, as mentioned previously, the number of complexes of Pt(II) in *trans*-conformation displaying cytotoxicity is relatively small. The molecular graphs of cisplatin, transplatin and the investigated complex of 3-aminoflavone are shown in Figure 4, and the values of electron density (ρ_BCP_), Laplacian of electron density (∇^2^ρ_BCP_) and of the kinetic (G_BCP_), potential (V_BCP_), and total energy densities (H_BCP_) in bond critical points (BCPs) are collected in Table 3. 

There is one very important feature of the results presented in Table 3 for Pt-N and Pt-Cl bonds. When one considers solely the values of electron density for these two types of bonding, it seems clear that electron density value in the BCP of the Pt-Cl bond in the 3-aminoflavone complex (0.0944 au) resembles the one found for transplatin (0.0957 au) and not the one found for cisplatin (0.1026 au). However, for Pt-N contacts, a reverse situation takes place, namely the electron density in the BCP for *trans*–Pt(3-af)_2_Cl_2_ (0.1074 au) is closer to the one found for cisplatin (0.1033 au) and not to the one found for transplatin (0.1160). An analogous situation takes place for other QTAIM parameters considered in this study and also for the bonding distances (see Table 3 for bond length values). The replacement of chlorido ligand(s) by water molecules is usually considered to be an activation process allowing cisplatin to display its cytotoxic properties [47] and Pt-Cl bonds are no longer present in Pt(II) complexes targeting the cellular DNA. Thus, it is possible that the cytotoxic properties of *trans*–Pt(3-af)_2_Cl_2_ (the trans isomer) are somehow connected with the fact that the properties of Pt-N bonds in *trans*–Pt(3-af)_2_Cl_2_ and cisplatin are similar to each other (but they are different from those found for transplatin of low cytotoxicity). Taking this into account, it is also worth considering the molecular electrostatic potential (MEPs) mapped onto molecular isosurfaces of these three platinum complexes. Electrostatic potential maps for these molecules are shown in Supplementary Figure S1.

As mentioned previously, cisplatin displays its cytotoxic properties after substitution of chlorido ligands by aqua ligands [48]. In the first step of the replacement process, the water molecule approaches the platinum center from the equatorial site of the complex, between the two ammine ligands. The aqua ligand binds to the platinum atom via Pt-O bond, and during the replacement process the oxygen atom possessing a partial negative charge points at positively charged platinum center between ammine ligands. The electrostatic potential value (mapped at the 0.01 au isosurface of electron density as shown in Figure S1 between the two ammine ligands is about 0.06 au, whilst for transplatin, the respective value between adjacent chlorido and ammine ligands is close to 0.02 au. Lower positive charge in the region between two ligands leads to lowering the electrostatic attraction of the oxygen atom during the replacement process for transplatin. Interestingly, for the title 3-aminoflavone complex, this value is about 0.06 au (as in the case of cisplatin), which means that in the activation process electrostatic attraction of the water oxygen atom by cisplatin and *trans*-Pt(3-af)_2_Cl_2_ is of similar strength but is significantly lower for transplatin.

### 2.3. Effect of Elevated Temperature of the Tested Formulations on the Survival of Caov-3 and OVCAR-3 Cells

The therapeutic use of hyperthermia is based on the assumption of a temperature increase up to 40–43 °C. The rationale for this therapy is based on the direct use of this method to kill cancerous cells at temperatures above 41–42 °C [14]. Hyperthermia is also used as a supportive therapy with various other treatments for cancer, such as radiotherapy and chemotherapy [7]. Some but not all studies have shown that that hyperthermia activates the immune system against cancer cells by increasing the release of heat shock protein (HSP) associated with cancer-specific antigen undergoing heat stress or dying cancer cells that are phagocysed by antigen-presenting cells (APC) [49].

As a result of heat, the cells produce heat shock proteins (HSPS), the proteins that are responsible for presenting the antigens and may affect immunogenicity [50]. The HSP protein family includes: HSP60, HSP70, HSP90, and sHSP low molecular protein [51]. HSPs are produced as a result of cell exposure to stress factors such as temperature, toxins, and non-oxygenation [52]. The heat shock proteins exhibit pro-and anti-apoptotic properties [53]. The heat shock protein HSP70 plays a role in thermotolerance, in hyperthermia an increase in production is observed [54,55]. The main role in thermoregulation plays the heat shock protein 70 (HSP 70) [56]. When hyperthermia is used in the treatment of ovarian cancer, a particularly interesting fact is that cancer cells are more sensitive to heat than normal cells [57,58]. Hyperthermia causes inhibition of cellular respiration and cell blockage in phase S is observed. After heating, the endovascular environment becomes acidous, unoxygenated and unnourished due to the likely damage to the vessels. The acidity of the environment also seems to increase the response of cancer cells to some medications at elevated temperatures [57]. Hyperthermia may potentiate the cytotoxic effects of cisplatinin vitro. High heat doses above 42 °C may partially reverse the resistance to cisplatin [59,60]. Resistance to cisplatin restricts the use of the medication at the clinic, it seems that combination of heat and cisplatin may be an interesting option to minimize this problem. According to Helma et al., elevated temperature of cytostatics appears to be an alternative promising therapy for patients with advanced cancer [61].

**Figure 5:**
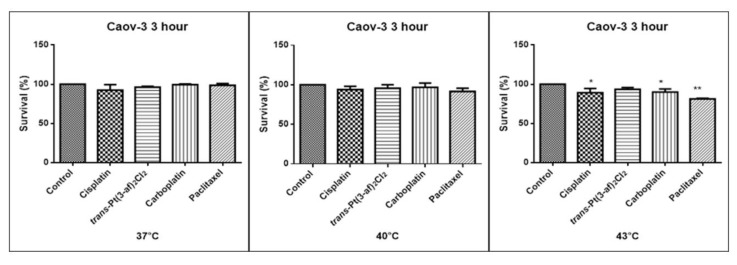
The effect of the elevated temperature of the tested formulations on the Caov-3 ovarian cancer line cells was determined by a MTT test. Data on the figures are presented as mean values obtained from three independent experiments * (*p* < 0.05), ** (*p* < 0.01).

**Figure 6:**
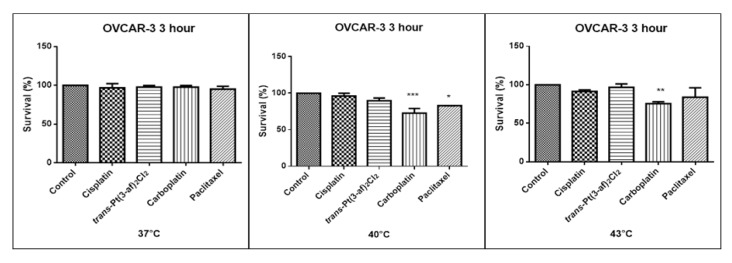
The effect of elevated temperatures on OVCAR-3 ovarian cancer line cells was determined by MTT test. Data on the figures are presented as mean values obtained from three independent experiments * (*p* < 0.05), ** (*p* < 0.01), *** (*p* < 0.001).

One of the aims of the study was to investigate the effects of elevated temperatures of the tested compounds (37 °C, 40 °C, 43 °C) on the survival of Caov-3 and OVCAR-3 cells. Temperature selection was made in accordance with literature data [62,63]. Decreased survivability was observed in cells exposed to elevated temperatures. There was no statistically significant decrease in the survival rate of the cells exposed to the elevated temperature of the tested preparations, i.e., 37 °C and 40 °C. A statistically significant decrease in the survival rate of the cells exposed to elevated temperatures at 43 °C was observed. Hyperthermia was shown to increase the cytotoxicity of the anticancer medications that were used. Reduced proliferation of cancer cells with chemotherapeutic agents was observed. It was shown that in OVCAR-3 and Caov-3 cells, the decrease in cell survival was not high at 37 °C.The analogue of the second-generation platinum, carboplatin, shows the best efficacy at the applied temperature (see Figure 5 and Figure 6). Not all anticancer medications visibly increased their activity at temperatures above 37 °C. Increased cytotoxicity may vary depending on temperature. Pioneering research from the years 1970s and 1980s showed that the use of heat induces cell death at temperatures above 43 °C in cell cultures and animal experiments, and sensitizes cancer cells to radiation and certain cytotoxic medications in the temperature range of 39–43 °C [64]. Cisplatin achieves the highest efficacy within 43 °C [65]. Literature data show that, in the case of cisplatin, it is most effective to heat the medication to 43 °C [59,60,65,66]. The present in vitro studies prove that cisplatin at elevated temperatures shows a beneficial cytotoxic effect. In this study, it was also shown that both OVCAR-3 and Caov-3 cells exposed to paclitaxel action at 43 °C exhibited the highest decrease in cell viability compared to cisplatin [67,68,69]. In the analysed study, OVCAR-3 cells were also observed to react with a decrease in cell viability after use of carboplatin at elevated temperatures between 40 °C and 43 °C [70]. It is worth to emphasize the new cisplatin analogue with 3-aminoflavone reduces cell vitality at elevated temperatures between 40 and 43 °C. Its effect on the line sensitive to cytostatics Caov-3 in 40 and 43 °C correlates with the action of cisplatin. In the case of the OVCAR-3 resistant line at 40 °C, the new compound is more effective, i.e., the number of living cells decreases compared to the action of cisplatin.

### 2.4. Effect of Elevated Temperature of Tested Formulations on Caov-3 and OVCAR-3 Cell Mortality

**Figure 7:**
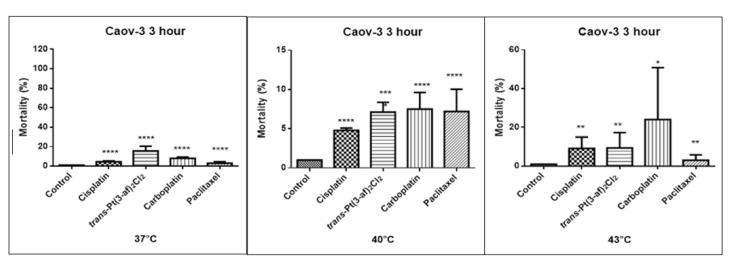
The effect of the elevated temperature of the tested preparations (43 °C) on the cells of the Caov-3 ovarian cancer line was determined by the LDH test. Data on the figures are presented as mean values obtained from three independent experiments * (*p* < 0.05), ** (*p* < 0.01), *** (*p* < 0.001), **** (*p* < 0.0001).

**Figure 8:**
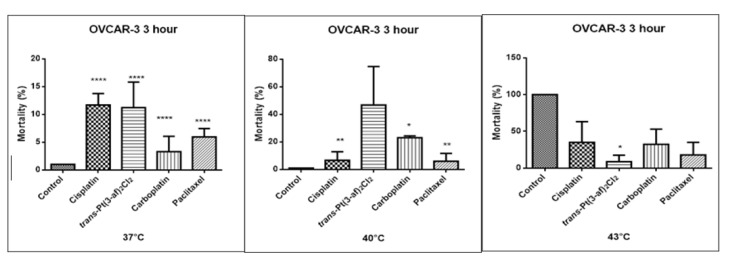
The effect of elevated temperatures on OVCAR-3 ovarian cancer cells was determined by the LDH test. Data on the figures are presented as mean values obtained from three independent experiments * (*p* < 0.05), ** (*p* < 0.01), **** (*p* < 0.0001).

One of the objectives of the study was to investigate the effect of elevated temperature of the test preparations (37 °C, 40 °C, 43 °C) on the mortality of Caov-3 and OVCAR-3 cells (see Figure 7 and Figure 8). Decreased survival of the cells exposed to elevated temperatures was reported. A statistically significant change in the activity of lactate dehydrogenase released into the nutrient medium, with an increase in the number of dead cells after 3 hours of incubation, was demonstrated. The highest amount of lactate dehydrogenase in a medium, proving the increase in the number of dead cells after 3 hours of incubation, was observed after exposure to carboplatin heated to 43 °C.

### 2.5. Assessment of the Effects of the Tested Formulations on the Expression of Selected Genes on Caov-3 and OVCAR-3 Cells

**Figure 9:**
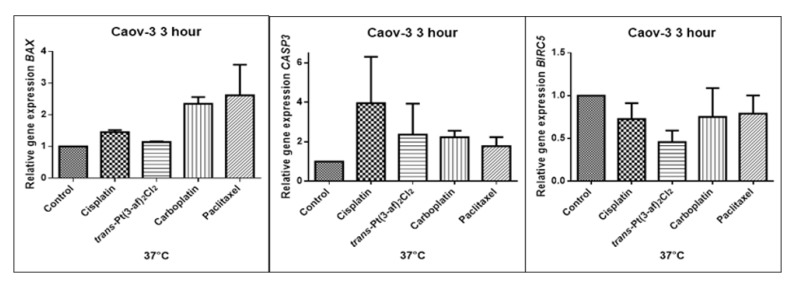
Level of gene expression of *BAX, CASP-3, BIRC5* in ovarian cancer cells Caov-3. The gene expression level was presented as a relative value compared to a control equal to 1.

**Figure 10:**
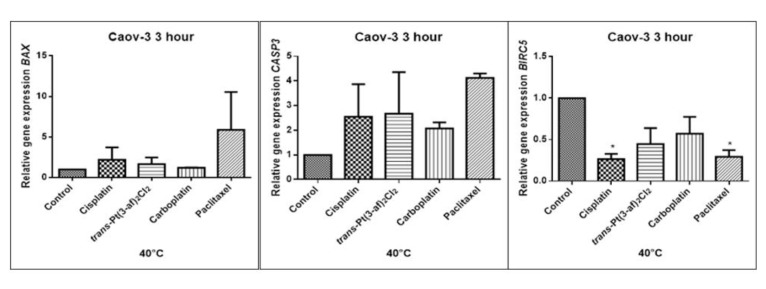
Level of gene expression of *BAX, CASP-3, BIRC5* in ovarian cancer cells Caov-3. The gene expression level was presented as a relative value compared to a control equal to 1. The mean values marked by * (*p* < 0.05) significantly differ statistically from the control.

**Figure 11:**
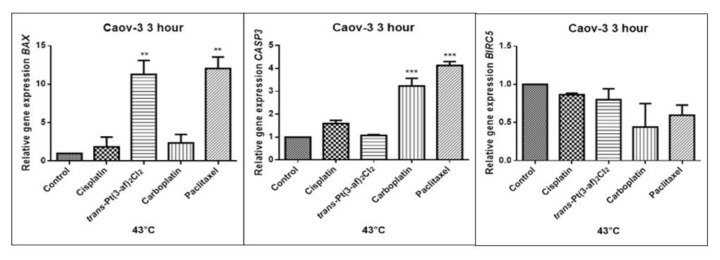
Level of gene expression of *BAX, CASP-3, BIRC5* in ovarian cancer cells Caov-3. The gene expression level was presented as a relative value compared to a control equal to 1. The mean values marked by ** (*p* < 0.01), *** (*p* < 0.001) significantly differ statistically from the control.

**Figure 12:**
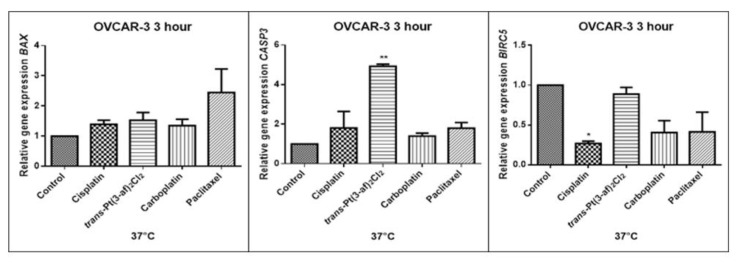
Level of gene expression of BAX, CASP-3, BIRC5 in ovarian cancer cells OVCAR-3. The gene expression level was presented as a relative value compared to a control equal to 1. The mean values marked by * (*p* < 0.05), ** (*p* < 0.01), significantly differ statistically from the control.

**Figure 13:**
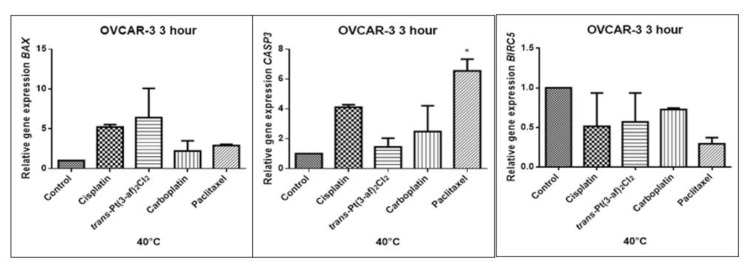
Level of gene expression of *BAX, CASP-3, BIRC5* in ovarian cancer cells OVCAR-3. The gene expression level was presented as a relative value compared to a control equal to 1. The mean values marked by * (*p* < 0.05) differ statistically significantly from the controls.

**Figure 14:**
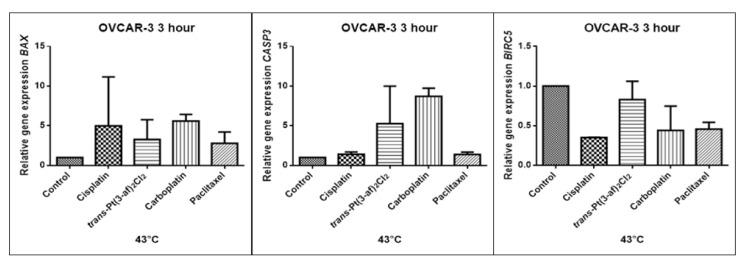
Level of gene expression of *BAX, CASP-3, BIRC5* in ovarian cancer cells OVCAR-3. The gene expression level was presented as a relative value compared to a control equal to 1.

In order to investigate the effect of the elevated temperature of the tested preparations (37 °C, 40 °C, and 43 °C) on the expression of apoptosis genes (*BAX, CASP3, BIRC5*), the Caov-3 and OVCAR-3 cells with selected concentrations (IC_50_) were incubated for 3 hours. The effect on the expression of pro-and anti-apoptotic genes in ovarian cancer cells was determined by the Real-Time PCR method. A change in gene expression profile was observed as a result of 3-hour incubation of cells with tested preparations (see Figure 9, Figure 10, Figure 11, Figure 12, Figure 13 and Figure 14).

The aim of cancer therapy is apoptosis. Inducing apoptosis is subject to test with use of new combinations of oncological therapies. Molecular analysis was therefore performed. The result of the study related to the gene of survivin is interesting. It is important to note that survivin is overexpressed in tumors and is not expressed at all in healthy tissues. Lowering the expression of survivin affects the mechanism of resistance to cytostatics. This is related to the activation of apoptosis. Gene *BIRC5* survivin decreased significantly as a result of the use of paclitaxel in the case of Caov-3 and use of carboplatin in the OVCAR-3 line. The survivin gene is a gene that is located in the mitotic apparatus, reflecting the important function it performs in the cell division process. The level of survivin expression is controlled by TP53 protein. In the case of DNA damage (e.g., due to the use of cytostatic at elevated temperature), the intracellular pathway TP53-survivin is activated. As a result, the expression of survivin and induction of apoptosis is reduced. Survivin is associated with cell cycle phases, during mitosis it travels to the mitotic spindle, which may indicate good treatment effectiveness with paclitaxel that cause impairment of the mitotic spindle. The decline in the expression of survivin is associated with an increase in apoptosis after the use of paclitaxel and cisplatin. Significantly studies have shown that the new platinum(II) compound *trans*–Pt(3-af)_2_Cl_2_ correlates similarly to cisplatin, at the same time lowering the expression of survivin. The decline in expression of survivin significantly enhances cell sensitivity to apoptosis. Molecular studies confirm that in 43 degrees in Caov-3 cells, the decline in the expression of survivin increases the antineoplastic activity of cisplatin and the new analogue shown in the MTT assay.

*Trans*–Pt(3-af)_2_Cl_2_ at 43 degrees in OVCAR-3 cells had the weakest cancer response obtained in the MTT test. This result confirms the molecular study, the survivin gene is in this case higher than after cisplatin administration. The expression ofthe *BAX* gene after exposure to platinum(II) complex with 3-aminoflavone at 43 °C on Caov-3 cells correlates with the expression after treatment with paclitaxel, which belongs to the gold standard of ovarian cancer treatment. Nevertheless, when the temperature of 40 °C is applied, it correlates with the action of cisplatin. This suggests that not every medication exhibits the same effect at any temperature, with regard to the change in gene levels. The increase in the expression of the *BAX* gene in OVCAR-3 cells was associated with decreased cell viability at 43 °C, with a new compound causing an increase in the expression of the *BAX* gene at 40 °C and decrease in cell survival. Silencingin Caov-3 cells of the expression of survivin after the use of carboplatin and paclitaxel at 43 °C resulted in cell death caused by hyperthermia.

Resistance to medications is the main cause of treatment failure and mortality in cancer patients. One of the mechanisms leading to overcoming resistance may be the use of hyperthermia. Hyperthermia can inhibit DNA repair, promote accumulation of medications and increase membrane permeability. It was found that the use of medicines at elevated temperatures could result in a change in the expression of apoptosis gene levels compared to untreated cells. In the present study, cytostatics with hyperthermia induced apoptosis in ovarian cancer cells OVCAR-3 and Caov-3 by increasing the expression of *BAX* and *CASP3* and reducing the expression of *BIRC5*. Many studies currently analyse the effects of elevated temperatures on cell survival in a cytostatic environment [71,72,73].

## 3. Materials and Methods

### 3.1. Cambridge Structural Database Search

The search of the Cambridge Structural Database (CSD, Ver. 5.40 release November 2018) [74] was performed with Conquest Ver. 2.0.1 program in order to obtain information about the known crystal structures displaying structural similarities to the cisplatin drug (*cis*-diamminedichloroplatinum(II) complex-Pt(NH_3_)_2_Cl_2_). In the search, platinum atoms were specified as possessing a formal positive charge “2+” and only the complex compounds of the coordinative number equal to 4 with only single bonds from platinum to nitrogen and chlorine atoms were taken into account. The search criteria were further limited to restrict the search to the highest-quality results: the crystal structures with R factors larger than 0.05, showing disorder, including numerical errors, or obtained from X-ray powder studies, were excluded from the analysis. 

### 3.2. Molecular Hirshfeld Surface Analysis

Molecular Hirshfeld surfaces and fingerprint plots based on the CIF files obtained from the CSD search were generated with Crystal Explorer 3.0 program [75] in order to analyze the scheme of intermolecular interactions around the platinum center. Hydrogen atom positions were normalized to standard bond lengths from neutron diffraction (C–H = 1.083 Å, O–H = 0.983 Å, N–H = 1.009 Å) [76] using the automatic procedures implemented in the program. The normalized contact distance (d_norm_) was mapped onto the Hirshfeld surfaces. The normalized contact distance used in the study is defined using the following formula: d_norm_ = [(d_i_–r_i_)/r_i_] + [(d_e_–r_e_)/r_e_], [77] where d_i_ and d_e_ are the distances between a chosen point of the Hirshfeld surface and, respectively, the nearest atom of the analyzed molecule (internal d_i_) and the neighboring molecule (external d_e_); r_i_ and r_e_ are van der Waals radii of the corresponding atoms. The results of mapping onto the molecular Hirshfeld surfaces are presented in this paper according to the standard red-white-blue coloring scheme: white color corresponds to intermolecular contacts of length close to the van der Waals separations, red-denotes shorter contacts of negative values of d_norm_, and blue corresponds to long contacts characterized by positive values of d_norm_. This relatively new method was effectively used to describe intermolecular interactions in various types of crystals [78,79,80,81].

### 3.3. Computational Methods

The structures of cisplatin, transplatin, and the title 3-aminoflavone complex were optimized at the B3LYP/def2-TZVPP level [82,83,84,85,86,87]. It should be stressed here that the def2-TZVPP basis set by definition includes pseudopotentials on platinum atoms accounting for relativistic effects. For stationary points a frequency analysis at the same level of theory was carried out in order to check whether the optimized geometries correspond to potential energy surface minima; no imaginary frequencies were found. The above calculations were performed using the Gaussian09 program [88]. For optimized geometries of the three platinum (II) complexes an analysis of the molecular electrostatic potential (MEP) and electron density distribution in the framework of the quantum theory of atoms in molecules (QTAIM) [89] was carried out. The QTAIM analysis provides several important quantities such as for example the electron density (ρ) or the Laplacian of the electron density (∇^2^ρ) calculated at specific points of the molecular space, the latter parameter describes local charge concentration and depletion. The QTAIM method is a powerful tool of structural chemistry allowing to characterize and define various types of bonding of both closed- and shared-shell character [90,91,92]. In the present study QTAIM calculations using the AIMAll program [93] were performed in order to obtain insight into the nature of interatomic bonding around the platinum center in the investigated systems. 

### 3.4. Cells and Reagents

Cell lines of ovarian cancer OVCAR-3 and Caov-3 were purchased at the American Type Culture Collection (ATCC, Manassas, VA, USA). OVCAR-3 ovarian cancer cells were cultured in RPMI 1640. Ovarian cancer cells Caov-3 were bred in DMEM. The medium was supplemented by 20% (OVCAR-3) and 10% (Caov-3) of fetal bovine serum and antibiotic solution.

### 3.5. Viability Assays

IC_50_ concentrations of tested medications were established in previous experiments and were added to cells in the logarithmic growth phase and then incubation lasted for 3 hours at temperatures 37–43 degrees Celsius [94], (Table 4). OVCAR-3 and Caov-3 cells were seeded on 24-well plates in a quantity of 20 × 103/500 μL of the medium a well. Cell growth was evaluated with the MTT test (3-(4,5-dimethylthiazol-2,5-diphenyltetrazolium bromide). At the end of the incubation the medium was removed and 5 mg/mL MTT (Sigma Aldrich, Darmstadt, Germany) was added. The absorbance of formazane was measured at a wavelength of 540 nm with background subtraction at 690 nm. A spectrophotometer for scanning microplates was used for measurements.

### 3.6. Cytotoxicity Assay

The LDH test (CytoSelect™ LDH Cytotoxicity Assay Kit, Cell Biolabs) was applied for evaluation of the cytotoxicity of the tested medications. OVCAR-3 and Caov-3 cells were seeded on 24-well plates in quantities of 20 × 10^3^/500 μL of medium per a well. After 24 hours of incubation in the wells, IC_50_ concentrations of tested compounds were added and incubated at elevated temperatures: 37 °C, 40 °C, 43 °C in an incubator for 3 hours. After 3 hours of incubation, 90 μL of the breeding medium was transferred to the 96-well plate in accordance with the protocol enclosed to the set and 10 μL of the reagent was added to the LDH catalysed reaction. Cells were incubated for 3 hours at 37 °C. After incubation, the coloured reaction product was measured using a plate reader from the BioTek company at a wavelength of λ = 450 nm. Control cells have not been treated.

### 3.7. Real-Time PCR Amplification Product Quantitative Analysis

The level of gene expression involved in the apoptotic process (Table 5) was determined by gene expression analysis by Real-Time PCR. Ovarian cancer cells needed to examine the level of apoptosis gene expression were seeded on bottles of 25 cm^2^ in the amount of 40 × 10^3^. After 24 hours, the tested compounds were administered and incubated at elevated temperatures for 3 hours. RNA isolation from ovarian cancer cells was performed according to the Chomczynski and Sacchi methods using the Trizol reagent (Thermo Fisher Scientific, Waltham, MA USA) [95]. The purity of the received RNA was determined by the spectrophotometric method on the Quawell UV-Vis spectrophotometer Q 5000 by measuring the absorbance at 260 nm and 280 nm wavelengths. The purity criterion for RNA was the absorbance value of 260/280, within 1.8–2.0. The reverse transcription reaction was performed using a set of High Capacity RNA-to-cDNA Kit (Thermo Fisher Scientific, Waltham, MA USA), according to the manufacturer’s protocol. The received cDNA was a Real-Time PCR matrix for the determination of the *BAX, BIRC5* and *CASP3* gene expression. The reaction mix contained: 50 ng of cDNA (0.5 μL), 5 μL of TaqMan Gene Expression Master Mix (Thermo Fisher Scientific, Waltham, MA, USA), 0.5 μL of probe TaqMan Gene Expression Assay (Thermo Fisher Scientific, Waltham, MA, USA) (Table 5) and 2.5 of water. Real-Time PCR reactions were carried out in the ViiA7 instrument (Thermo Fisher Scientific, ViiA™ 7 Software v. 1.1 Waltham, MA, USA). The gene expression results obtained were compared with the reference gene *ACTB.* The relative level of gene expression was calculated using Method2^−ΔΔ*C*t^. Gene expression level was presented as relative values compared to the untreated control equal 1.

### 3.8. Statistical Analysis

The statistical analysis was performed using GraphPAD Prism6 computer program of the GraphPad Software Inc company. The results of the conducted experiments were analysed by the Bonferonni test to determine statistically significant changes between the control groups and the groups treated with studied compounds. A significance level below 0.05 (* *p* < 0.05; ** *p* < 0.01; *** *p* < 0.001; **** *p* < 0.0001) was considered statistically significant.

## 4. Conclusions

In an attempt to design more effective drugs, a better understanding of the molecular mechanisms underlying theantitumour effects of platinum(II) compounds is needed. Hence, the molecular structure and intermolecular interactions of the title complex are compared with other cisplatin analogues (CSD). A possible correlation between the structure and anticancer activity of trans–Pt(3-af)_2_Cl_2_ was suggested. The chemical part of our study on *trans*–Pt(3-af)_2_Cl_2_ has focused on analysis of the crystal structures and intermolecular interactions around the platinum center of molecules. Summarizing the results of Hirshfeld surface analysis, it may be concluded that the areas above and below the platinum atom (with respect to the plane of the complex) play an important role in stabilization molecular packing in the crystal state. Moreover, the obtained results suggest that platinum atom could be rather easily available for water molecules and the first step of hydration process may be connected with formation of O-H…Cl- hydrogen bonds between the water molecule and the chlorido ligand. Interestingly, the results of analysis of the molecular electrostatic potential indicate that in the activation process, electrostatic attraction of the water oxygen atom by cisplatin andby the title 3-aminoflavone complex is of similar strength. It is also possible that cytotoxic properties of *trans*–Pt(3-af)_2_Cl_2_, being similar to the cytotoxic properties of cisplatin, are associated with the fact that the properties of Pt-N bonds in both compounds display close resemblance to each other. 

In the biological part of article, particular attention was paid to the intensification of anticancer effects. Cisplatin, *trans*–Pt(3-af)_2_Cl_2_ and other cytostatics were examined at elevated temperatures. Hyperthermia in combination with chemotherapy is not a novel treatment for cancer. However, the mechanism of action of combination therapy is not fully understood. In the present study, ovarian cancer cell lines such as OVCAR-3, Caov-3 with a different clinical pedigree and carcinogenic pathomechanism were used. Cell viability was examined and the expression of some of the key genes associated with apoptosis (Caspase-3, BAX and Survivin) was determined. After the application of hyperthermia, significant apoptotic genes were shown to decline. Based on the experiments conducted in the present study real possibilities to increase the activity of cytostatics including cisplatin with elevated temperatures were demonstrated. Considering different pedigree of ovarian cancer is key when designing chemotherapy regimens. Elevated temperature of cytostatics (40 °C, 43 °C) increases the anticancer activity of the examined medications. Hyperthermia was shown to increase the sensitivity of cells to platinum medications. 

Noteworthy is the fact that the *trans*–Pt(3-af)_2_Cl_2_ can be used to successfully treat ovarian cancers with different pedigree like the gold standard medications, which is confirmed by the change in expression of key apoptotic genes after exposure to a new compound. Therefore, it seems possible that the newly synthesized compound may be effective in ovarian cancer therapy. The intensification of effect with hyperthermia opens up new possibilities in terms oftreatment, because elevated temperature affects the variable level of gene expression, and thus damage under the influence of cytostatics cell is directed to the apoptosis route.

## Figures and Tables

**Table 1:** Percentage of intermolecular contacts involving platinum atom resulting from Hirshfeld surface analysis of cisplatin analogues.

Refcode	Pt…Pt	Pt…H	Pt…All	Cl…H	Pt…Pt/Pt…All	Pt…H/Pt…All
*trans*						
MONVIW	-	-	-	8.5	-	-
RIWCEG	0.5	0.3	0.8	11.5	0.63	0.38
VOHBAW	-	1.5	1.9	10.3	-	0.79
*cis*						
BERDAE	1.0	2.6	4.2	36.1	0.24	0.62
BERDEI	0.3	2.7	3.1	33.4	0.10	0.87
CCENPT01	2.5	1.6	4.1	38.7	0.61	0.39
CUKRAB01	1.8	3.6	5.4	43.5	0.33	0.67
DIVXOV	-	1.3	1.8	20.5	-	0.72
FITFUJ	0.9	1.3	2.5	21.2	0.36	0.52
KIPSEH	-	2.8	2.9	30.0	-	0.97
LAYZEQ	0.4	1.6	2.0	18.3	0.20	0.80
LIXTOB	-	0.1	0.1	25.1	-	1.00
PEXTIV	0.9	1.5	2.9	24.9	0.31	0.52
PIFGIU	-	-	-	19.4	-	-
SUDMIN02	1.5	1.4	3.2	32.4	0.47	0.44
TAJTED	1.2	1.2	3.0	25.9	0.40	0.40
UCIZUC	2.1	1.4	3.6	34.2	0.58	0.39
YIDVUD	0.1	0.6	0.8	21.9	0.13	0.75

**Table 2:** Comparison of selected intramolecular bond lengths (in Å) and angles (in degrees) of *trans*–Pt(3-af)_2_Cl_2_complex.

Bond	Crystal Structure	Optimized Structure
Pt1-N3	2.064(5)	2.100
Pt1-Cl1	2.298(1)	2.340
O1-C2	1.366(7)	1.360
O1-C9	1.374(7)	1.363
C2-C3	1.355(8)	1.358
C2-C11	1.479(8)	1.473
C3-N3	1.443(7)	1.433
C3-C4	1.451(8)	1.461
C4-O4	1.231(7)	1.228
C4-C10	1.460(8)	1.461
N3-Pt1-Cl1	92.7(1)	95.7
Pt1-N3-C3	121.7(4)	121.0
C2-O1-C9	120.0(4)	121.6
O1-C2-C3	121.4(5)	120.2
O1-C2-C11	111.2(5)	111.3
C3-C2-C11	127.3(5)	128.4
C2-C3-N3	122.0(5)	125.0
C2-C3-C4	122.2(5)	122.1
N3-C3-C4	115.7(5)	112.9
O4-C4-C3	122.1(5)	120.7
O4-C4-C10	123.0(5)	124.2
C3-C4-C10	114.8(5)	115.4
C3-C2-C11-C12	−56.5(8)	−38.3
C4-C3-C2-C11	−179.4(5)	−172.5
Pt1-N3-C3-C2	88.0(6)	89.5

**Table 3:** Selected geometric and QTAIM parameters for cisplatin, transplatin and *trans*–Pt(3-af)_2_Cl_2_complex: bond lengths (d, in Å), electron density (ρ_BCP_, in e Å^−3^), Laplacian of electron density (∇^2^ρ_BCP_, in e Å^−5^), kinetic (G_BCP_ in hartree Å^−3^), potential (V_BCP_ in hartree Å^−3^) and total energy density(H_BCP_ in hartree Å^−3^) at the respective BCPs.

Complex	Bond Type	d	ρ_BCP_	∇^2^ρ_BCP_	G_BCP_	V_BCP_	H_BCP_
*cis*-Pt(NH_3_)_2_Cl_2_	Pt-N	2.109	0.1033	0.3584	0.1210	−0.1526	−0.0317
	Pt-Cl	2.305	0.1026	0.1947	0.0878	−0.1271	−0.0393
*trans*-Pt(NH_3_)_2_Cl_2_	Pt-N	2.059	0.1160	0.3962	0.1387	−0.1789	−0.0401
	Pt-Cl	2.333	0.0957	0.2031	0.0847	−0.1187	−0.0341
*trans*-Pt(3-af )_2_Cl_2_	Pt-N	2.100	0.1074	0.3355	0.1193	−0.1550	−0.0357
	Pt-Cl	2.340	0.0944	0.1996	0.0830	−0.1163	−0.0333

**Table 4:** IC_50_ values of the investigational medicinal preparations.

Drugs	Caov-3	OVCAR-3
Cisplatin	10 µM	50 µM
*Trans*-Pt(3-af)_2_Cl_2_	10 µM	50 µM
Carboplatin	10 µM	25 µM
Paclitaxel	10 µM	5 µM

**Table 5:** Taq Man probes used for REAL-Time PCR reactions.

Gen	Probe Number
BAX	Hs 00180269-m1
BIRC5	Hs 00153353-m1
CASP3	Hs 00234387-m1
ACTB	Hs 01060665-g1

## Supplementary Materials

Supplementary materials can be found at https://www.mdpi.com/1422-0067/21/6/2116/s1.
