# Supplementary material for: Simple Trans-Platinum Complex Bearing 3-Aminoflavone Ligand Could Be a Useful Drug: Structure-Activity Relationship of Platinum Complex in Comparison with Cisplatin
Source: Int J Mol Sci. 2020 Mar 19;21(6):2116. doi: 10.3390/ijms21062116 (PMC7139614; doi:10.3390/ijms21062116)
Supplement: Supplementary file 1 [file ijms-21-02116-s001.pdf]

# Supporting information

**Table S1.** Mean values of geometric parameters around the Pt center in the selected platinum complexes: bond lengths (in Å) and angles (in degrees).

| refcode | Pt-Cl | Pt-N | Cl-Pt-N | Cl-Pt-Cl | N-Pt-N |
|---|---|---|---|---|---|
| MONVIW | 2.298 | 2.064 | 87.3 | 180 | 180 |
| RIWCEG | 2.305 | 2.005 | 89.1 | 178.2 | 176.5 |
| VOHBAW | 2.303 | 2.024 | 83.8 | 180 | 180 |
| BERDAE | 2.306 | 2.031 | 92.6 | 92.1 | 82.7 |
| BERDEI | 2.318 | 2.021 | 91.0 | 93.5 | 84.4 |
| CCENPT01 | 2.319 | 2.033 | 91.7 | 93.2 | 83.1 |
| DIVXOV | 2.307 | 2.025 | 91.7 | 92.8 | 83.8 |
| FITFUJ | 2.308 | 2.025 | 92.1 | 92.7 | 83.0 |
| LAYZEQ | 2.312 | 2.032 | 86.8 | 92.6 | 90.2 |
| LEFFAD | 2.301 | 2.039 | 91.3 | 92.0 | 83.2 |
| LIXTOB | 2.315 | 2.043 | 88.1 | 93.8 | 90.0 |
| PEXTIV | 2.325 | 2.031 | 90.8 | 94.8 | 83.6 |
| PIFGIU | 2.307 | 2.051 | 87.3 | 93.0 | 92.8 |
| SUDMIN02 | 2.323 | 2.085 | 88.2 | 92.5 | 90.0 |
| TAJTED | 2.316 | 2.034 | 91.9 | 93.7 | 82.6 |
| TUPQIE | 2.312 | 2.051 | 92.9 | 90.8 | 83.8 |
| UCIZUC | 2.329 | 2.040 | 86.9 | 93.0 | 93.0 |
| YIDVUD | 2.299 | 2.049 | 88.9 | 91.3 | 91.3 |

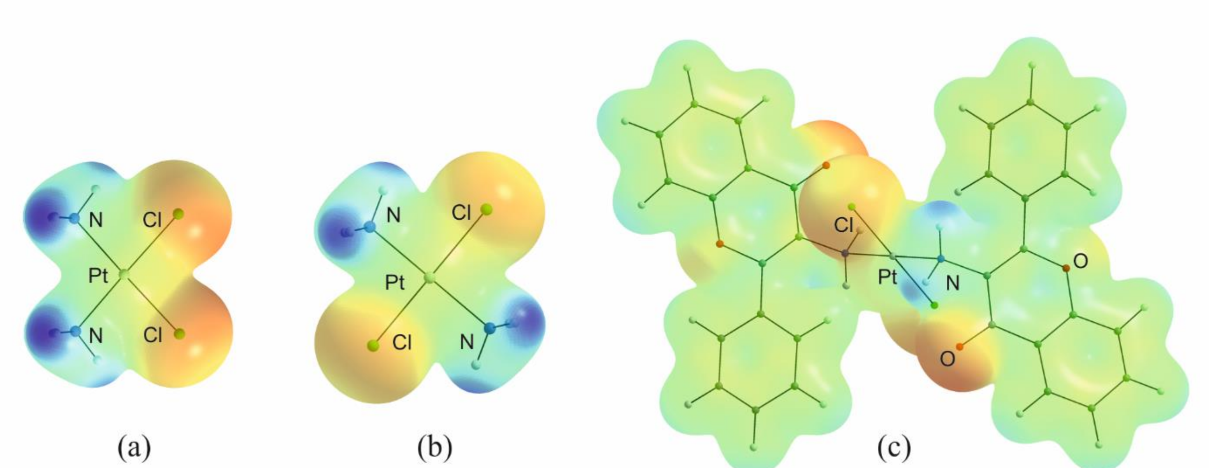


(a) (b) (c)

**Figure S1.**Molecular electrostatic potential maps with the colored scale corresponding to values ranging from −0.10 (red) to +0.20 au (blue) mapped onto the 0.01 au isosurface of electron density: cisplatin (**a**), transplatin (**b**) and ***trans*-Pt(3-af )$_2$Cl$_2$** (**c**) calculated at the B3LYP/def2-TZVPP level.
